# Game-based training of selective voluntary motor control in children and youth with upper motor neuron lesions: protocol for a multiple baseline design study

**DOI:** 10.1186/s12887-021-02983-8

**Published:** 2021-11-11

**Authors:** Annina Fahr, Andrina Kläy, Larissa S. Coka, Hubertus J. A. van Hedel

**Affiliations:** 1 Swiss Children’s Rehab, University Children’s Hospital Zurich, Mühlebergstrasse 104, 8910 Affoltern am Albis, Switzerland; 2 Children’s Research Center, University Children’s Hospital Zurich, Steinwiesstrasse 75, 8032 Zürich, Switzerland; 3 Institute for Biomechanics, ETH Zurich, Leopold-Ruzicka-Weg 4, 8093 Zurich, Switzerland

**Keywords:** Neurorehabilitation, Single-case design, Interactive computer play, Cerebral palsy

## Abstract

**Background:**

Impairments of selective control of joint movements can have consequences for many activities of daily life, but there are only a few interventions to improve selective voluntary motor control (SVMC). We have developed a treatment option to specifically enhance SVMC exploiting the advantages of interactive computer play technology. It targets SVMC by training selective activation of a muscle or a selective joint movement while it provides immediate feedback about involuntary muscle activations/movements at an (unwanted) joint. This study aims to investigate the effectiveness of this game-based intervention to enhance SVMC in children and youth with upper motor neuron lesions.

**Methods:**

We will conduct a randomized, non-concurrent, multiple baseline design study. Patients aged between 6 and 20 years with reduced SVMC due to an upper motor neuron lesion will be included. During the baseline phase of random length, participants will attend their regular intensive rehabilitation program, and in the intervention phase, they will additionally complete 10 therapy sessions (à 40 min) of the game-based SVMC training. The primary outcome will be a short SVMC assessment conducted repeatedly throughout both phases, which quantifies movement accuracy and involuntary movements. Changes in clinical SVMC measures, muscle strength, cortical excitability, motor control of the inhibited/unwanted movement, and functional independence will be assessed as secondary outcomes. We will use a mixed-effect model to determine the change in the course of the primary outcome when the intervention is introduced, and we will compare changes between phases for secondary outcomes with paired tests.

**Discussion:**

This study will provide first evidence whether SVMC can be improved with our game-based training. The single-case design takes into account the individualization required for this intervention, and it can help to address the challenges of intervention trials in our setting.

**Trial registration:**

German Clinical Trials Register: DRKS00025184, registered on 28.04.2021.

**Supplementary Information:**

The online version contains supplementary material available at 10.1186/s12887-021-02983-8.

## Background

Loss of selective voluntary motor control (SVMC) is one common sign of upper motor neuron lesions in children, e.g., due to traumatic brain injury, stroke, or spastic cerebral palsy (CP) [1]. SVMC has been defined as “the ability to isolate the activation of muscles in a selected pattern in response to demands of a voluntary posture or movement” [2]. Clinically, reduced SVMC can manifest in impaired motor control and a multitude of involuntary movements, i.e., unintended movements that co-occur with the performance of a voluntary task [3]. These include mass flexion/extension patterns and synergies of muscle activation (i.e., obligatory grouped multi-joint movements) or mirror movements (i.e., simultaneous identical movements on the contralateral side) [4, 5].

These involuntary muscle activations and movements after a lesion to the upper motor neuron are addressed to different neurophysiological origins. Synergistic co-activation of muscles is suggested to result from a compensatory reliance on the extrapyramidal rubrospinal tract, which is responsible for patterns of coupled muscle activation [1]. The occurrence of mirror movements is thought to involve bilateral cortical activation due to insufficient interhemispheric inhibition and spared ipsilateral corticospinal projections [6].

Loss of SVMC is listed as a core impairment for children with CP [7] and can negatively influence other body functions and activities. For example, selective motor control showed a stronger relation to gross motor function abilities compared to other common impairments of children with spastic CP, like muscle weakness or spasticity [8, 9]. Reduced SVMC is also associated with a less favorable course of gross motor function in longitudinal evaluations [10, 11]. Furthermore, impaired SVMC has a negative impact on the walking abilities of children with spastic CP, e.g., gait velocity [12]. For the upper extremities, children with unilateral spastic CP who exhibit mirror movements need more time for bimanual activities compared to peers without mirror movements [5]. Thus, a lack of SVMC can interfere with functional movements of daily life activities and contribute to limitations of the children’s independence and participation.

Although the importance of selective motor control has been recognized in several cross-sectional studies, only a few interventions have specifically aimed to improve it [13]. For example, a robot-assisted ankle movement training implemented into a computer game was used to train graded ankle dorsi- and plantarflexion [14]. In another study, selective muscle activation was trained with a commercial video game controlled by surface electromyography signals to reinforce desired muscle activity and reduce co-contraction of the agonist-antagonist muscle pair [15]. Although both interventions could show promising results in preliminary studies so far, these training approaches can address only one specific aspect of SVMC (i.e., improving joint movement control or reducing agonist-antagonist co-contraction).

To fill this gap, we have recently developed an intervention specifically targeting the improvement of SVMC that intends to cover all features of reduced selective control. It consists of a game played in a virtual environment coupled to a technology-assisted interface to track joint movements. This allows to train accurate joint movement control, while it simultaneously provides immediate feedback about the occurrence of involuntary movements (via an alarm sound). Advantages of using interactive computer play technology are that playful environments can enhance motivation to enable large numbers of repetitions and the possibility to improve movement performance based on augmented feedback. Both are important aspects of motor learning during rehabilitation [16]. In a small pilot trial, five children with impaired SVMC completed five training sessions (lasting 45 min each) with the game-based intervention to enhance SVMC. It proved to be feasible and motivated participants to practice, but effects on SVMC were not examined [17]. Therefore, the primary aim of this study is to investigate the effectiveness of this game-based intervention in improving SVMC in children and youth with upper motor neuron lesions in a multiple baseline single-case design. Our hypothesis is that ten sessions of game-based training of SVMC concomitant to intensive rehabilitation improve SVMC more than standard rehabilitation alone.

Secondary aims of this study are to investigate a) whether the effectiveness of the intervention is related to factors like age, diagnosis, or muscle strength and clinical SVMC measures at baseline; b) the time to response, i.e., amount of training needed until a meaningful improvement in SVMC can be expected; c) the effect of the intervention on clinical measures of SVMC, muscle strength, cortical excitability, motor control of the inhibited movement, and functional independence; d) the association of changes in SVMC with changes in muscle strength or cortical excitability; and e) whether potential changes in SVMC, muscle strength, and functional independence are maintained three months after the intervention.

## Methods/design

This study will be conducted in compliance with the Declaration of Helsinki and was approved by the cantonal ethics committee of Zurich (BASEC Nr. PB 2021–00791). The trial was listed in the German Clinical Trials Register (DRKS00025184, registered on 28.04.2021) before patient recruitment started.

### Study design

**Fig. 1:**
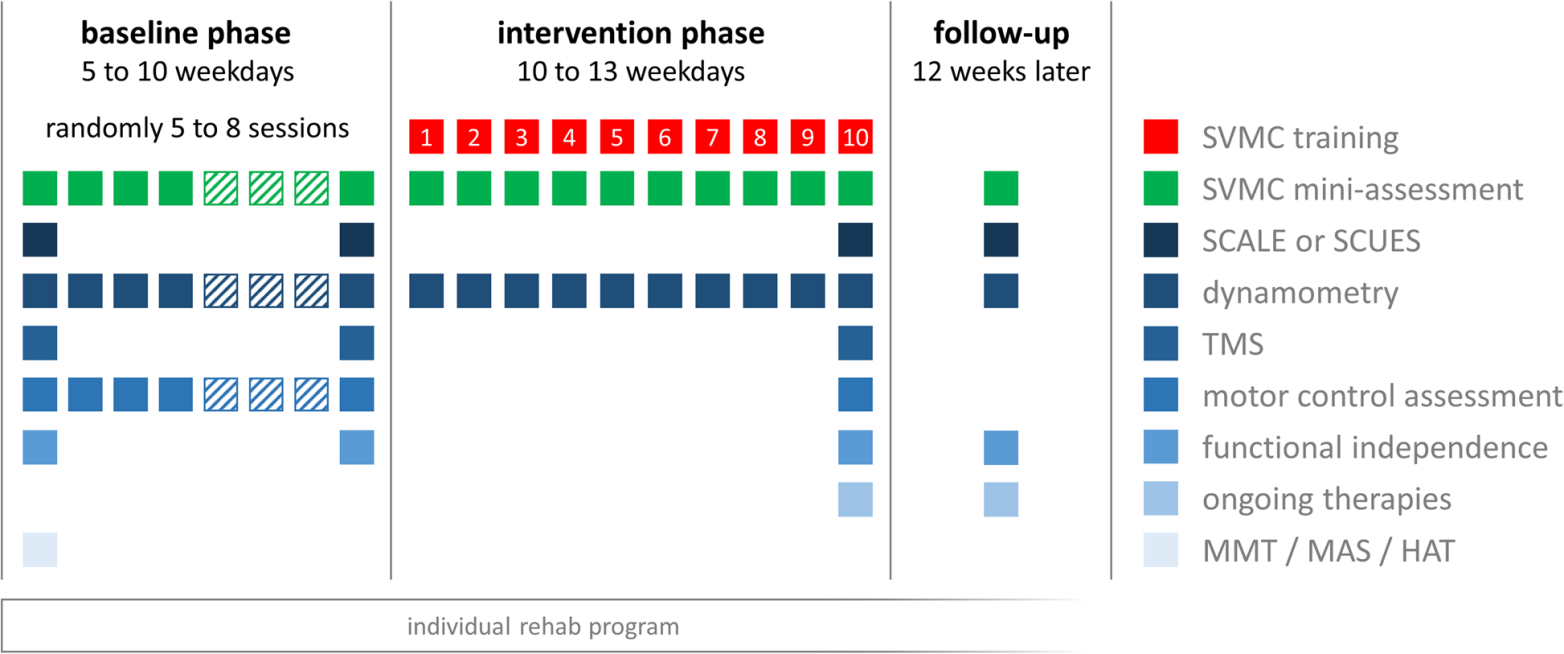
Flow diagram of the study. The baseline phase will encompass randomly between 5 to 8 assessment sessions. Comprehensive outcome measurements will be conducted during the first and last appointment. For the other occasions, only a short assessment of selective control, muscle strength and motor control of the joint showing involuntary co-movements or co-activations is planned. During the intervention phase, participants will receive 10 sessions of game-based SVMC training. The short assessments (only SVMC and strength) will be continued after each treatment session, and the full assessment battery will be repeated at the end of the intervention as well as 12 weeks after completion. The study will run concurrently to the participant’s individual rehabilitation at the Swiss Children’s Rehab. Abbreviations: SVMC: selective voluntary motor control, SVMC mini-assessment: short game-based assessment to repeatedly measure selective control, SCALE: selective control assessment of the lower extremity, SCUES: selective control of the upper extremity scale, TMS: transcranial magnetic stimulation, MMT: manual muscle test, MAS: modified Ashworth scale, HAT: Hypertonia Assessment Tool

We will use a randomized, non-concurrent, multiple baseline design across participants to investigate the efficacy of our game-based intervention to improve SVMC. The study will consist of a baseline phase of random length, i.e., 5 to 8 short assessment sessions distributed over 5 to 10 weekdays. The assessment sessions will include the measurement of the primary outcome (a short game-based SVMC assessment) and dynamometry of the target muscle group. During the intervention phase, participants will receive 10 treatment sessions of 40 min of our game-based SVMC training during 10 to 13 weekdays, while the short assessment will be continued (Fig. 1). Participants will train selective control of one individually determined target movement or muscle group, while trying to reduce the occurrence of involuntary movements or muscle activations around another predefined joint. Further assessments (see outcome measures) will be conducted at the beginning of the baseline phase, at the transition from the baseline to the intervention phase, at the end of the intervention phase, and at a follow-up 12 weeks after completion of the intervention.

The study will run concurrently to intensive multimodal rehabilitation at the Swiss Children’s Rehab, which can consist of physical-, occupational- or speech and language therapy, sports therapy including endurance or strength training or sports groups, and/or robotics arranged according to the individual needs of the patient. A given number of assessment time points but variable time periods for each phase allow some flexibility to fit the children’s schedules.

### Eligibility criteria and recruitment

Participants fulfilling the following inclusion criteria will be eligible for the study: a) acquired or congenital brain injury that caused an upper motor neuron lesion; b) aged between 6 and 20 years; c) impaired SVMC of the target joint indicated by scores 0 or 1 for the lower extremity movement in the Selective Control Assessment of the Lower Extremity (SCALE) or scores 1 or 2 for an upper extremity movement in the Selective Control of the Upper Extremity Scale (SCUES); d) Manual Muscle Test (MMT) score ≥ 2 of the target joint; e) pain-free movement of the involved joints; and f) ability to understand and follow two-step commands, e.g., close your eyes and clap your hands, to guarantee the ability to handle two instructions as during the intervention, i.e., move one joint without moving another one.

Exclusion criteria encompass: a) ataxia or primary dyskinetic movement component (dystonia, athetosis, chorea) in the involved joints due to the brain injury; b) surgery or treatment with Botox during the last 3 months in one of the involved joints; c) uncorrected visual and/or auditory limitations that hinder playing the game; d) skin lesions that prevent the correct placement of sensors or electrodes; e) inability to play the game for any other reason; and/or f) non-compliance with the instructions. For transcranial magnetic stimulation (TMS) assessments, the following additional exclusion criteria apply: g) epileptic seizure within the last 2 years; h) implanted electronic devices; i) severe or recent heart disease; and/or j) any other reason for exclusion from TMS indicated by the treating physician. Participants with contraindications for TMS will be excluded from TMS assessments but are still eligible to participate in the study.

For this study, we will recruit patients of the Swiss Children’s Rehab during daily clinical practice. Participants and their legal guardians will be provided with oral and written information about the study, and written informed consent will be obtained from all the legal guardians and the participants prior to enrolment into the study.

### Participant characteristics

We will describe the study population by the clinical characteristics age, diagnosis, and the Gross Motor Function Classification System (GMFCS) level, or the Manual Ability Classification System (MACS) level. The GMFCS standardizes the classification of gross motor function, emphasizing trunk control and walking ability of children diagnosed with cerebral palsy [18]. Children at GMFCS level I perform all the activities neurologically intact children of the same age can, allowing for slight limitations in speed and quality of movements. Children with GMFCS level V exhibit difficulties in head and trunk control in most positions or achieving any voluntary control of movement at all. The MACS classifies how children with cerebral palsy handle objects in daily activities [19]. Level I means that the child can handle objects easily and successfully, whereas children at level V do not handle objects at all. For children with CP, medical professionals routinely assess GMFCS and MACS levels in the Swiss Children’s Rehab. For this study, we will also use this classification for children with other diagnoses.

To better describe the target joint and the joint where involuntary movements should be reduced, experienced therapists will apply the MMT and the Modified Ashworth Scale (MAS). The MMT measures function and strength of individual muscles/muscle groups based on the effective performance of a movement in relation to the forces of gravity and manual resistance on a six-point ordinal scale [20]. The MAS describes spasticity by rating resistance to passive joint movement with varying degrees of velocity on a level between 0 (no increase in muscle tone) to 4 (rigid in flexion or extension) [21]. We will determine the type of hypertonia of the trained extremity with the Hypertonia Assessment Tool (HAT) [22, 23].

### Randomization and blinding

We will use an urn randomization scheme to determine the number of sessions (between 5 and 8) in the baseline phase [24]. The urn design incorporates probabilities of assignment that adapt according to the degree of assignment imbalance. Thus, with this method, allocation is weighted towards a balanced distribution.

Blinding of the participants to the study phase is not possible due to the nature of the intervention. Clinical assessments (SCALE/SCUES) will be videotaped and evaluated after completion of the study by uninvolved assessors blinded to the time point of the recordings. For assessments that need immediate evaluation and cannot be videotaped, the assessor will be unaware of previous results by using a new score sheet. The instrumented assessments are less prone to bias because they do not depend on a person’s judgment.

### Intervention

**Fig. 2:**
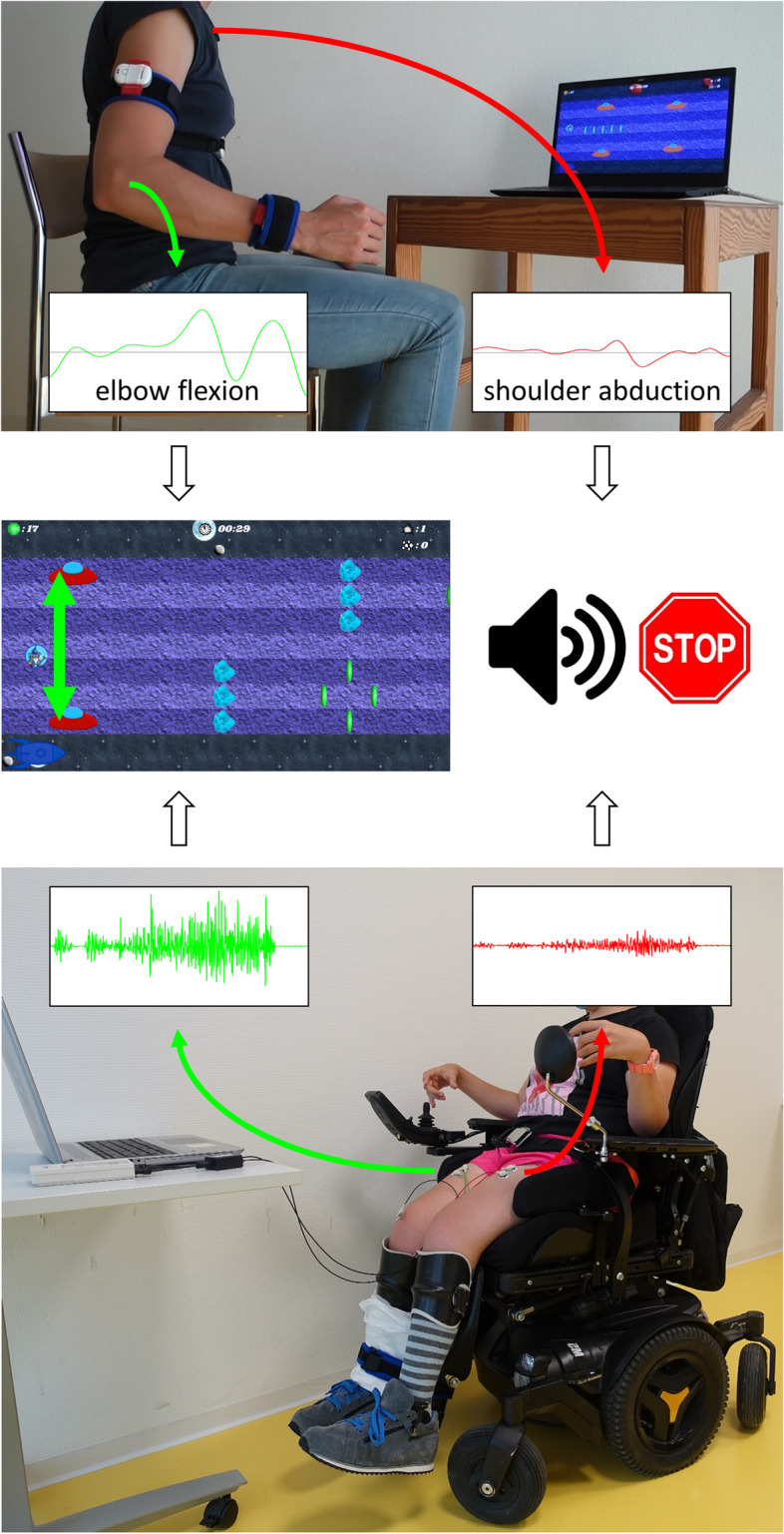
Setup of the game-based intervention. The avatar can be controlled by joint movements (e.g., elbow flexion as in the upper picture) or by muscle activations (right *M. rectus* *femoris* as in picture at the bottom). Involuntary movements (e.g., shoulder abduction) or muscle activations (left *M. rectus* *femoris*) trigger the feedback signal to make the player aware of their occurrence and to reduce them

The game-based intervention to train SVMC consists of a custom game environment and technology-assisted interface (Fig. 2). It trains selective movement or muscle activation at the target joint and reducing involuntary movements or muscle activations that occur at another (unwanted) joint. Details on the development of the game are described elsewhere [17].

During the game, an avatar is steered up and down by moving the target joint or activating the target muscle group. The goal of the game is to collect coins and avoid certain obstacles. As soon as an involuntary movement or muscle activation occurs, an auditory augmented feedback signal makes the player aware of the occurrence of involuntary movements (Fig. 2). The signal volume is graded to the extent of involuntary movements or muscle activation. Furthermore, the avatar’s speed gradually slows down, also relative to the extent of involuntary movements. This response to involuntary occurring activations or movements was implemented to make gameplay temporarily easier. It should enable the player to focus again on the game and reduce the occurrence of co-movements because these often appear in conditions of increased effort [3]. The game environment is structured by a hierarchical arrangement of levels of increasing difficulty that are progressively unlocked, similar to commercial video games. Nine levels together constitute a game “world”, each with a thematically different design. At the onset of each level, 3 challenges (e.g., collecting a certain amount of coins) are presented. Progression to the next world requires that 80% of the challenges of the current world are fulfilled. Game progress can be saved in individual profiles, so the players will resume where they stopped the previous session. Throughout the intervention, we will give standardized tutorial-like step-by-step instructions to guide the participant through the game.

The game can either be controlled by joint movements or muscle activation, both serving to train SVMC (Fig. 2). For the first control approach, joint angles are captured with the ArmeoSenso rehabilitation system (Version 1.0, Hocoma AG, Volketswil, Switzerland) by three inertial measurement units and transferred to the game computer via the User Datagram Protocol. Up- and downward movements of the avatar are caused by moving the target joint in an upwards and downwards direction, respectively. Movements of the joint that should not move activate the “warning” feedback signal. The reference sensor of the ArmeoSenso will be attached with an elastic band around the chest. We will use Velcro straps to attach the two distal sensors to the trained extremity. For the upper extremities, the sensor positions will be laterally on the upper arm and wrist like a watch (i.e., the standard application). For the lower extremities, one sensor will be positioned laterally on the lower leg and the other one laterally on the edge of the foot. Standardized body positions for the training are outlined in the Additional file 1. The ArmeoSenso system has first to be calibrated according to the manufacturer’s software before we will calibrate our game system to the participant’s active range of motion (ROM) of the target and the involuntary movement joint. The game will require movements within 80% of the calibrated range, omitting the top and bottom 10%.

For the second approach to controlling the SVMC game, surface electromyographic (sEMG) signals of the target and involuntarily activated muscle groups are recorded using a varioport device (Becker Meditec, Karlsruhe, Germany) at a sampling frequency of 1000 Hz. Electrodes (Kendall H124SG) will be placed on the muscle belly of the two involved muscles and a reference electrode on a bony process. The sEMG signals are transferred via Bluetooth to the game computer, where they are filtered (exponential smoothing with a smoothing factor of 0.003 followed by taking a moving average with window size 10 frames). Increasing and lowering the activation of the target muscle causes the avatar to move upwards or downwards, respectively. Activity in the muscle that should remain inactive triggers the feedback signal. The target joint will be fixed in standardized positions (see Additional file 1) with Velcro straps and a customized board with loops screwed on it to enable isometric muscle activation. Isometric training was chosen since not restraining movement caused pain after only a short playing time in the pilot trial because the joint was mostly held in a position at the end of the ROM. To calibrate the system, we will record a 4 s baseline when participants relax and two maximal voluntary contractions (MVC). A baseline-corrected activity level of 20% MVC is required to reach the top position in the game, and the lowest position corresponds to 2% MVC (i.e., 10% of played range). If the control is too sensitive (i.e., due to a low MVC level resulting in a small signal range), the upper limit will be increased to maximize the range while keeping the effort reasonable.

Movements that can be trained comprise shoulder abduction, elbow flexion, wrist extension, finger flexion, knee extension, and ankle dorsiflexion. Participants will train selective control of one individual target movement while trying to reduce movements in one other joint. This combination will be selected according to the participants’ individual goals and in consultation with their therapist. Based on experience from the pilot trial [17], more proximal target joint movements (shoulder, elbow, and knee joint) will be trained with the ArmeoSenso unless in combination with a reduction of mirror movements (which the ArmeoSenso is unable to track). Movement at distal joints will be trained with the sEMG based system.

Appointments in the intervention phase will last 45 min, such that they fit the therapy schedule of the clinic. Forty minutes are planned for treatment with the game-based intervention, including preparations and pauses if needed, and the last 5 min will be reserved for outcome assessments. Movement scientists trained and experienced in the treatment of patients with movement disorders will deliver the intervention.

### Primary outcome

**Fig. 3:**
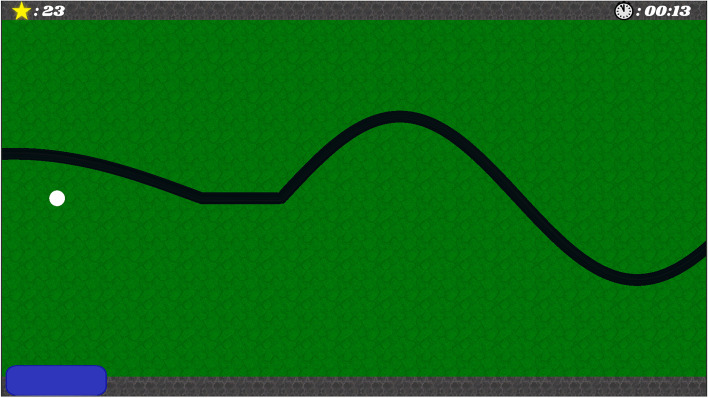
Screenshot from the mini-assessment. It displays the black target line, which the participant has to follow with the white dot avatar. The target line describes up and downward curves of varying amplitudes and frequency. The avatar is steered by movements of the target joint or activity in the target muscle. The blue bar in the bottom left corner displays the extent of involuntary movements additionally to the auditory feedback signal

Outcome measures for single-case designs have to be suitable for frequent repeated assessment throughout the baseline and intervention phase. Thus, the primary outcome will be SVMC, measured with a short game-based assessment (called ‘mini-assessment’). The principle to measure SVMC with this mini-assessment is similar to a previously developed assessgame [25, 26], and we will use the same setup as for the intervention. The participant has to perform target joint movements of different speeds and amplitudes to follow a target line describing up and downward curves on the screen with an avatar (Fig. 3). Similar to the intervention, participants receive immediate auditory feedback about the occurrence of any involuntary movements.

Before starting, participants will receive standardized instructions to aim for staying on the target line and for minimizing the feedback signal as much as possible. Throughout the assessment, no comments or feedback will be given. One test trial will last 30 s, and the assessment will be repeated 3 times with at least 30 s rest in between the trials. The outcomes will be averaged to get more stable results.

During the intervention phase, the test will be conducted immediately after the training. In the baseline phase and at follow-up (i.e., no preceding training), the assessment session will start with a 75 s try-out period in an environment resembling the mini-assessment. In this period, the target line comprises straight lines at different positions in the beginning and curves through the whole calibrated ROM in the end. Thereby, we can verify that the steering is working properly, and participants can familiarize themselves with the task to reduce initial learning effects.

The outcome metrics of the mini-assessment encompass first, the accuracy of the target movement (root mean squared error between avatar and target line) and second, the occurrence of involuntary movements (in % of the calibrated ROM). Both outcomes will be transformed to percentages relative to the mean of all baseline points for each participant. In the end, these values will be averaged to yield the primary dependent variable.

### Secondary outcomes

Most secondary outcome measures will be applied at distinct time points at the beginning and end of each study phase to assess pre- to post-changes for each study phase as well as at the follow up (see Fig. 1 for details). Muscle strength and motor control abilities of the “unwanted” movement will be assessed at similar time points as the primary outcome.

#### Clinical measures of SVMC

We will conduct the SCALE [27] or the SCUES [28], depending on whether a lower or upper extremity target joint will be trained. These clinical assessments require the participant to perform reciprocal movements at various joints: hip flexion/extension, knee flexion/extension, ankle dorsiflexion/plantarflexion and eversion/inversion, and toes flexion/extension for the lower extremities, and shoulder abduction/adduction, elbow flexion/extension, forearm pronation/supination, wrist flexion/extension, and finger flexion/extension for the upper extremities. The assessment will be videotaped for blinded evaluation. SVMC is scored on a three (SCALE) or four-point (SCUES) scale for each movement. Variables of interest are the score of the target joint, the summed score of the target joint side, and the total score. These two clinical tools have been shown to be valid and reliable to assess selective control of the lower/upper extremities in our target population [27–30].

#### Muscle strength

As strength was shown to be related to SVMC [31], we will assess the muscle strength of the target joint with dynamometry. We will use a hand-held dynamometer (microFET 2, Hoggan Scientific, Salt Lake City) to measure the force produced. We will conduct the test in standardized positions (see Additional file 1) to increase reliability and use the average value received from three repetitions allowing 30 to 60s rest in between trials. Upon the go-signal, participants should push as hard as they can against the dynamometer for 5 s while the assessor counts back the seconds.

#### Cortical excitability

We will use a standardized protocol to determine the motor threshold and the latency and amplitude of the motor evoked potential (MEP). A single-pulse TMS will be performed using a MagPro X100 Magnetic Stimulator (MagVenture A/S, Farum, Denmark) in combination with a Keypoint G3 Workstation (DANTEC Medical A/S, Skovlunde, Denmark). Electrodes will be placed on the muscle belly of the muscle that is also the target muscle group of the intervention. For all measurements, a MFC-B65 Butterfly figure-of-eight coil will be used to stimulate the primary motor cortex. If a lower extremity muscle group is trained, the coil will be positioned just lateral to the midline, close to Cz (Vertex), contralateral to the target muscle [32]. If an upper limb muscle group is trained, the coil will be positioned close to C3h (left) or C4h (right) [33]. The coil will be moved in small increments to find the location with the largest response (i.e., hot spot). Participants will wear a bathing cap and this location will be marked on it. We will use a biphasic single-pulse stimulus, as these are more effective than monophasic pulses, with a pulse duration of 200 μs. The stimulus threshold will be expressed as the percentage of maximal stimulator output that evokes an MEP amplitude of at least 50 μV in approximately 50% of 10 consecutive stimuli (i.e., motor threshold). The stimulation intensity will be set at 1.2 times the motor threshold. We will perform five stimulation repetitions to determine the parameters latency and amplitude of the MEP in the averaged signal. On the second and third TMS test occasions, the procedure will be repeated. Again the stimulation threshold is determined and noted for comparison. Independent from the new threshold level, the stimulations to determine MEP latency and amplitude are performed at the same level of stimulator output as performed during the first measurement. TMS measurements will be conducted in separate appointments.

#### Motor control of the joint showing involuntary co-movements or co-activations

We want to explore whether our training intervention also affects movement control of the joint that is showing involuntary muscle activations/movements. Our exploratory hypothesis is that learning to inhibit muscle activity/movement by means of the SVMC game could lead to improved control of that muscle/joint. To study this, participants will perform the mini-assessment with the joint that will become trained to stay “inactive” during the game. For this research question, we will only evaluate the accuracy of the movement (i.e., no feedback on involuntary movements for this test). Repeated testing will be conducted during the baseline phase and at the end of the intervention to keep the burden for the participants low. Procedures will be the same as described for the primary outcome, including the try-out phase.

#### Functional independence

To assess the children’s functional independence in daily life activities, we will use the domains mobility (for the lower extremities) and self-care (for the upper extremities, excluding bladder and bowel items) of the Functional Independence Measure for children (WeeFIM®). The WeeFIM® measures activity on the performance level and rates independence on a scale from 1 (total assistance needed) to 7 (complete independence, performs task timely and safely) [34]. In the Swiss Children’s Rehab, it is assessed by trained and certified nurses on a regular basis.

#### Potentially confounding ongoing therapies

We will record concomitant regular therapies the participants attend over the course of the study between the first and last mini-assessment session of each phase. The corresponding therapist(s) will be asked whether improving SVMC was part of their therapy sessions.

#### Measures describing the intervention

Intervention sessions will be described by the active training time when the participant was actually playing, excluding the setup and any rests taken between levels, which will be automatically logged. We will further record any events during the training, like software errors or modifications to the setup.

### Sample size calculation

We approximate the number of participants needed with calculations based on a general linear model and later correct for the fact that measurements from one participant are not independent observations (clustering). In a general linear model, 78.4 observations are sufficient to detect a medium to large effect (r = 0.4, R^2^ = 0.16) [35] with a power of 90%, α = 0.05 and 3 degrees of freedom for the numerator (two fixed effects and one random effect), calculated with the pwr.f2.test function of the R package ‘pwr’ [36].

The correction, also called the design effect, accounts for the degree of variance inflation that attributes to clustering (several measurements from one participant). The design effect equals 1 + (*m* − 1) ∗ *ρ*, where m is the number of data points per cluster (i.e., participant) and ρ is the intraclass correlation within clusters [37]. We estimate ρ = 0.18 for the percentage score combining the accuracy and involuntary movement outcome based on unpublished data from our pilot study with six participants. In this trial, the mini-assessment was conducted three times before the first training and once before and after the following 4 training sessions. According to the formula, the design effect thus equals 3.80, assuming that m = 16.5 data points will be recorded for a person on average (i.e., expected value for randomly between 5 and 8 assessments in the baseline phase and 10 during the intervention).

Multiplying 78.4 with the design effect yields the total number of observations needed, which is then divided by 16.5 observations per person. Allowing a dropout rate of 10%, we will need 20 participants.

### Statistics

#### Primary research aim

The statistical analyses will be carried out with R statistical package [38]. The primary analysis of the efficacy will include a (hierarchical) mixed-effect model for the variable combining the accuracy and involuntary movement outcome. On a first level, the scores of the individual participant will be modeled by a set of predefined predictors, i.e., the session number (time), and the interaction of the session with the treatment phase. On a second level, the model parameter estimates will be allowed to vary between participants (random effects). We will account for the serial dependency of the data with a first-order autoregressive correlation structure of within-subject errors. The parameter of primary interest will be the interaction of time with the treatment phase, hence the change in trend when the intervention is introduced. Depending on the distribution and shape of the trend of the data, linear or additive mixed models will be used. Additionally, the same analysis will be conducted for the accuracy and involuntary movement outcome separately to investigate whether they show comparable effects.

For a patient-level analysis, a similar regression model will also be run for each individual participant.

#### Secondary research aims

We will extend the primary model to analyze whether there exist interactions between the main parameter of interest (interaction of session with treatment phase) and secondary or descriptive outcomes. These other predictors encompass participant characteristics, active training time, muscle strength, and clinical SVMC measures of the target joint at baseline. Changes in muscle strength will also be analyzed with a mixed-model with the same parameters described above for the primary outcome.

We will calculate Kaplan-Meier curves that model the estimated probability of nonresponse by the number of intervention sessions and determine the median time to response (smallest number of sessions at which the probability drops to 0.5 or below). The criterion for response will be an improvement by 10% compared to the baseline level in the game-based mini-assessment of SVMC, represented by two consecutive data points in the intervention phase exceeding this threshold. As studies about meaningful changes in SVMC are not available yet, we estimated the response threshold via the standard error of measurement of several assessments of SVMC. One standard error of measurement corresponds roughly to changes of 10%.

For clinical SVMC measures, cortical excitability, and functional independence, changes during the baseline and intervention phase will be compared using paired t-tests or robust alternatives. We will account for the variable length of these phases by analyzing changes relative to the length of the corresponding phase. The length of each phase will be defined as the number of therapy days in between the first and last mini-assessment of each phase. The days of the first or last assessments themselves will also be counted if the first session was in the morning or if the last session was in the afternoon. We will test in the same way for differences in the frequency of other therapies participants attended between baseline and intervention phase. To analyze changes in the accuracy of motor control of the joint/muscle that will be trained to remain inactive, we will check whether the post-intervention score lies within the 95% prediction interval of the baseline scores.

To analyze the association of changes in SVMC and muscle strength, we will correlate the regression coefficients for the interaction of the session and treatment phase from the strength and the SVMC model of each individual participant. For TMS outcomes, we will further correlate pre- to post-intervention differences with changes in SVMC measures. Follow-up data will be analyzed by paired comparisons (t-test or robust alternative) between post-intervention and follow-up assessments (to test whether the level is maintained) and between the measurement directly before the intervention and at follow-up (to test whether the level is still higher than before starting the intervention).

## Discussion

This protocol paper describes the design of a randomized multiple baseline single-case design study to investigate the effectiveness of a novel game-based intervention to improve SVMC in children with upper motor neuron lesions.

Single case designs have been used in educational research for many years and are emerging in research concerning children with CP [39]. Repeated assessment of the outcome throughout distinct phases of a study is a key element of single-case experimental designs, as well as the concept that participants act as their own control [40–42]. These designs emphasize the individual cases and aim to describe and understand the variability between participants [43]. Group-design studies, like randomized controlled trials that are still considered the gold standard for evaluating treatment effects, are, for example, less suitable for small and heterogeneous samples [44]. For this reason, Romeiser-Logan et al. [45] argue that such research designs could be particularly suitable for pediatric rehabilitation. Using a single-case design also helps to handle the individualization of our intervention in terms of selecting the trained movement and the system to control the game. The application of a reversal design with withdrawal phases would not be suitable for this setting where carry-over effects are expected yet even aimed for [42]. Although increasing the length of the phases would be desirable (i.e., a higher number of data points, more treatment sessions), it has to be balanced against the length of the children’s stay at the clinic.

A limitation of the current study design is that it only investigates the effect of additionally providing our game-based SVMC training to regular rehabilitation but does not allow to compare the intervention to an equally dosed alternative treatment. However, this design was chosen because it will be the first time an SVMC training that targets both accurate movement control and a reduction of involuntary movements is investigated, and thus, this study can provide a first proof of principle. Moreover, the game-based SVMC intervention will only make up a small part of the therapy program the participant will receive during the study period. The intensive rehabilitation program running in the background of the study could affect the treatment effects. We might only see small differences between the baseline and the intervention phase because the regular rehabilitation program is included in both phases. However, we assume we will be able to see treatment effects because we focus very specifically on SVMC, and improving SVMC is rarely a therapeutic goal in other therapies [46]. Furthermore, we provide the intervention with a high frequency, i.e., the treatment sessions will be distributed over a short period. Apart from the frequency, the total dosage is comparable to the protocols of the few studies that aimed at improving aspects of SVMC so far, which applied training intensities between 4 and 9 h of treatment over 5 or 6 weeks [14, 15, 47, 48]. We account for the effects of the concomitant therapies as well as for spontaneous recovery in patients with subacute acquired brain injuries by comparing the trend of changes between the baseline and intervention phase or the changes relative to the length of the phases. Besides analyses on the group level, we will also look at each participant individually to address the heterogeneity of the study population.

This study will also serve to assess the responsiveness and determine the clinically meaningful change of two previously developed SVMC assessments [25, 26, 49]. These investigations were not included in the assessments’ preliminary psychometric testing due to the lack of an intervention specifically aiming to improve SVMC. The sEMG based similarity index recorded during the SCALE or SCUES and the assessgame will be conducted additionally before and after the intervention phase. We will assess meaningful change in SVMC using a global rating scale. The participant’s physical or occupational therapist (for lower or upper extremity target joints, respectively) will rate how SVMC of the participant has changed throughout the intervention on a five-point Likert scale: ‘much better’, ‘somewhat better’, ‘unchanged’, “somewhat worse’, ‘much worse’. We will also ask the participants themselves whether they have the impression, they can move their target joint more selectively. If therapists and particularly the participants would consider changes meaningful, this study would provide first evidence of efficacy and relevance of this game-based intervention to improve impairments in SVMC in children and adolescents with an upper motor lesion.

## Supplementary Information

**Additional file 1.** Measurement Protocol. Outlines the standardized body position used for the game-based intervention and dynamometry assessment.

## Data Availability

Not applicable.

## Abbreviations

CP: Cerebral palsy
GMFCS: Gross Motor Function Classification System
HAT: Hypertonia Assessment Tool
MACS: Manual Ability Classification System
MAS: Modified Ashworth Scale
MEP: Motor evoked potential
MMT: Manual Muscle Test
MVC: Maximum voluntary contraction
ROM: Range of motion
SCALE: Selective Control Assessment of the Lower Extremity
SCUES: Selective Control of the Upper Extremity Scale
sEMG: Surface electromyography
SVMC: Selective voluntary motor control
TMS: Transcranial magnetic stimulation
WeeFIM®: Functional Independence Measure for children
