# Supplementary material for: Game-based training of selective voluntary motor control in children and youth with upper motor neuron lesions: protocol for a multiple baseline design study
Source: BMC Pediatr. 2021 Nov 11;21:505. doi: 10.1186/s12887-021-02983-8 (PMC8582135; doi:10.1186/s12887-021-02983-8)
Supplement: Supplementary file 1 — Additional file 1. Measurement Protocol. Outlines the standardized body position used for the game-based intervention and dynamometry assessment. [file 12887_2021_2983_MOESM1_ESM.docx]

**Additional material**

**Measurement Protocol**

Body positions when the game is controlled by joint movements captured with the ArmeoSenso

| **Target movement** | **Body position** |
| --- | --- |
| Shoulder abduction | Sitting on a chair, elbow 90° flexed, forearm neutral |
| Elbow flexion | Sitting on a chair, shoulder adducted, forearm neutral |
| Wrist extension | Sitting on a chair, shoulder adducted, elbow 90° flexed, forearm pronated |
| Knee extension | Sitting on a high bench, hips 90° flexed, lower legs hanging freely |
| Ankle dorsiflexion | Sitting on a high bench, hips and knees 90° flexed, feet hanging freely |

Body positions when the game is controlled by muscle activation

| **Target movement** | **Main muscle** | **Body position** | **Fixation** |
| --- | --- | --- | --- |
| Shoulder abduction | M. deltoideus pars acromialis | Sitting on a chair, shoulder adducted, elbow 90° flexed, forearm resting on the thigh | Strap around the distal upper arm and trunk |
| Elbow flexion | M. biceps brachii | Sitting on a chair, shoulder adducted, elbow 90° flexed, forearm supinated (if possible) and lying on the attachment board on a table | Strap around the forearm just proximal to the wrist joint |
| Wrist extension | M. extensor carpi radialis | Sitting on a chair, shoulder adducted, elbow 90° flexed, forearm pronated and lying on the attachment board on a table | Strap over the hand just proximal to the metacarpal phalangeal joints |
| Finger flexion | M. flexor digitorum superficialis | Sitting on a chair, shoulder adducted, elbow 90° flexed, forearm neutral and lying on the table, holding a rod in the hand | - |
| Knee extension | M. rectus femoris | Sitting on a high bench, hips and knees 90° flexed, lower limbs leaning against the attachment board, feet hanging freely | Strap around the lower leg just proximal to the malleoli |
| Ankle dorsiflexion | M. tibialis anterior | Sitting on a chair, hips and knees 90° flexed, feet resting on the attachment board lying on the floor | Strap around the foot just proximal to the metatarso-phalangeal joints |

Gravity eliminated body positions for dynamometry

| **Target movement** | **Body position** | **Stabilization by assessor** | **Position of dynamometer** |
| --- | --- | --- | --- |
| Shoulder abduction | Supine, shoulder 45° abducted, arm neutral | Shoulder | Just proximal to the lateral epicondyle of the humerus on the lateral surface of the upper arm |
| Elbow flexion | Supine, shoulder adducted, elbow 90° flexed, forearm neutral or supinated (same position as during intervention) | Shoulder | Just proximal to the radial styloid process on the palmar/anterior or radial surface of the forearm |
| Wrist extension | Supine, shoulder adducted, elbow 90° flexed, forearm pronated | Forearm | Just proximal to metacarpal phalangeal joints on the palmar surface of the hand |
| Finger flexion | Sitting on a chair, shoulder adducted, elbow 90° flexed, forearm neutral | - | Participants clench the hand dynamometer |
| Knee extension | Sitting on a high bench, hips and knees 90° flexed, feet hanging freely, hands can be used for trunk stabilization | - | Between a strap around the leg of the bench and the participant’s lower leg, just proximal to the malleoli on the anterior surface of the lower leg |
| Ankle dorsiflexion | Supine, hips and knees 90° flexed, lower leg resting on a stool, ankle relaxed/resting position | Knee and lower leg | Just proximal to the metatarso-phalangeal joints on the dorsal surface of the foot |
